# [Corrigendum] Silibinin inhibits the migration and invasion of human gastric cancer SGC7901 cells by downregulating MMP‑2 and MMP‑9 expression via the p38MAPK signaling pathway

**DOI:** 10.3892/ol.2024.14753

**Published:** 2024-10-17

**Authors:** Shuming Lu, Zhuqing Zhang, Meiru Chen, Chunyan Li, Lina Liu, Yan Li

Oncol Lett 14: 7577–7582, 2017; DOI: 10.3892/ol.2017.7080

Subsequently to the publication of the above paper and a Corrigendum that has already been published (doi./10.3892/ol.2021.12676) to address the issue of data that had been duplicated in Figs. 1, 2 and 4, it has come to light that there was a further data panel in Fig. 4C that was overlapping with the published version of Fig. 2C that was not included in that corrigendum. The fully corrected version of Fig. 4, now showing the corrected data for the ‘+Silibinin, -SB203580’ experiment in Fig. 4C (second panel on the left), is shown below. The authors regret that this additional error was not included in the original corrigendum, and apologize to the Editor of *Oncology Letters* and to the readership for the additional inconvenience caused.

## Figures and Tables

**Figure 4.**
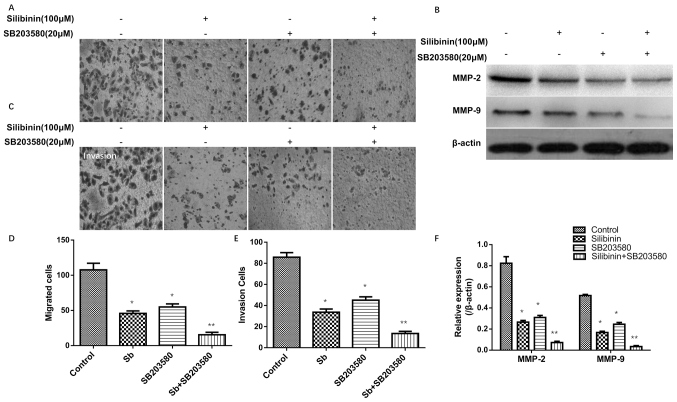
Effects of silibinin alone or in combination with SB203580 on cell migration and invasion. (A) Cell migration following treatment with silibinin alone or in combination with SB203580 (magnification, ×200). (B) The expression levels of MMP-2 and MMP-9 were (B) detected by western blot analysis. (C) Cell invasion following treatment with silibinin alone or in combination with SB203580 (magnification, ×200). Histograms present the (D) migrated cells, (E) invasive cells and (F) the quantified expression levels of MMP-2 and MMP-9. Control cells were treated with complete medium only. *P<0.05, **P<0.01 vs. control. MMP, matrix metalloproteinase; Sb, silibinin.

